# Autophagy mediates serum starvation-induced quiescence in nucleus pulposus stem cells by the regulation of P27

**DOI:** 10.1186/s13287-019-1219-8

**Published:** 2019-04-15

**Authors:** Bin Li, Chao Sun, Jing Sun, Ming-hui Yang, Rui Zuo, Chang Liu, Wei-ren Lan, Ming-han Liu, Bo Huang, Yue Zhou

**Affiliations:** 1 Department of Orthopedics, Xinqiao Hospital, Army Medical University, Chongqing, 400037 China; 2 Department of Orthopedics, Liaocheng People’s Hospital, Liaocheng, 252000 Shandong China

**Keywords:** Autophagy, Quiescence, Nucleus pulposus stem cell, P27

## Abstract

**Background:**

Adult stem cells exist in a quiescent state (G0) within the in vivo niche; the loss of quiescence often leads to a decrease in the number and function of adult stem cells, impairing tissue regeneration and repair. Endogenous repair by nucleus pulposus-derived stem cells has recently shown promising regenerative potential for the treatment of intervertebral disc degeneration (IDD). However, the number and function of nucleus pulposus stem cells (NPSCs) declined throughout the process of IDD. This effect may have a specific relationship with quiescence. However, the biology of the quiescent NPSCs has not been reported.

**Methods:**

First, we established an in vitro model for NPSC quiescence with serum starvation. The induction of G0 was confirmed by flow cytometry analyses of dual staining with Hoechst 33342 and Pyronin Y, immunofluorescent staining with Ki67 and Western blot analysis of P27 expression. NPSCs were cultured under serum starvation conditions for a long time period (21 days). To examine the functional phenotype of quiescent NPSCs, the cells were reactivated with 10% serum and differentiated into osteogenic and chondrogenic lineages in vitro. The number of colony-forming units was also estimated. To elucidate the role of autophagy in the quiescence of NPSCs, we activated and inhibited autophagy in starved cells with rapamycin and chloroquine, respectively. Then, the expression of P27 was evaluated by Western blot analysis, and the immunofluorescence of Ki67 was assessed. Finally, we assessed the role of P27 siRNA in NPSC quiescence by flow cytometry analyses and 5-ethynyl-20-deoxyuridine incorporation assays under normal and serum-starved conditions.

**Results:**

NPSC quiescence was induced by 48 h of serum starvation, and they maintained quiescence for up to 21 days. Upon reactivation with serum, the quiescent NPSCs re-entered the cell cycle and exhibited enhanced clonogenic self-renewal, osteogenic differentiation and chondrogenic differentiation potentials compared to control NPSCs under normal culture conditions. We also found that autophagy underlay serum starvation-induced NPSC quiescence. Further study demonstrated that autophagy mediated the quiescence of NPSCs by regulating P27.

**Conclusions:**

Serum starvation efficiently induces quiescence in NPSCs. Quiescent NPSCs maintain stem cell properties. Our study reveals that autophagy plays a role in maintaining NPSC quiescence and that autophagy mediates the quiescence of NPSCs by regulating P27. We conclude that the induction of quiescence in cultured NPSCs provides a useful model for the analysis of mechanisms that might be relevant to the biology of NPSCs in vivo.

## Background

Most mammalian adult tissues contain adult stem cells, which exist in a reversible and non-dividing state known as quiescence or G0 in uninjured tissues. Quiescence is critical for tissue-specific stem cells to maintain normal tissue homeostasis. The quiescent state protects adult stem cells from proliferative pressure as well as from damaging niche factors. Previous studies demonstrated that the loss of quiescence often leads to the premature exhaustion of haematopoietic stem cells and neural stem cells in p21-null mice [1, 2]. The disruption of quiescence in muscle stem cells leads to impaired muscle regeneration and repair [3]. Recently, a series of in vitro models of quiescence have been established, including serum starvation, contact inhibition, suspension culture and culture on soft polyacrylamide substrate [4, 5]. These in vitro models provide simple, readily scalable and reproducible systems for understanding the biological characteristics of G0 adult stem cells.

Evidence has been found regarding the existence of stem cells in the nucleus pulposus of intervertebral discs (IVDs). Activating these endogenous progenitor cells could be an attractive strategy for enhancing IVD regeneration [6]. However, recent studies demonstrated that the number and functions of NPSCs declined over time during the process of degeneration and ageing [7, 8]. The mechanism of these pathological changes is unclear. Given the critical role of quiescence in adult stem cells, we speculate that quiescence may participate in the exhaustion and declined regeneration of NPSCs. However, nothing has been reported describing the biology of G0 NPSCs.

Different molecular mechanisms of adult stem cell quiescence have been demonstrated, including the involvement of both cell-intrinsic factors, such as the P53 and RB proteins and CDK inhibitors (P21, P27 and P57), and cell-extrinsic factors, such as Notch signalling. However, the molecular control of quiescence is still largely unknown. Thus, a better understanding of the mechanisms of quiescence is required.

Autophagy is a process in which cellular components such as proteins and damaged mitochondria are engulfed by autophagosomes and delivered to lysosomes to be degraded and recycled in order to maintain cellular homeostasis. In response to environmental stress, quiescent cells rely on autophagic processes for survival [9–11]. The induction of autophagy seems to be very important in the regulation of stem cell quiescence. Most recent studies have indicated that autophagy plays a key role in maintaining quiescence and stemness in adult stem cells [12, 13]. In the blood system, autophagy actively suppresses metabolism by clearing active, healthy mitochondria to maintain quiescence and stemness [12]. Basal autophagy is essential to maintain the quiescent state of satellite cells. The failure of autophagy causes aged satellite cells to transition from normal quiescence into an irreversible senescent state by the loss of proteostasis and increased mitochondrial dysfunction and oxidative stress [13]. However, the role of the molecular control of autophagy in the regulation of quiescence still remains to be fully elucidated. In NPSCs, whether and how autophagy maintains quiescence is largely unknown. In the present study, we aimed to understand the biology of quiescent NPSCs using an in vitro model of quiescence and investigate the role of autophagy in maintaining NPSC quiescence.

## Methods

### Ethics statement

This study was conducted in accordance with the ethical standards set by the Declaration of Helsinki. The methods of this study were approved by the Ethical Committee of Xinqiao Hospital and met the NIH guidelines for the care and use of laboratory animals.

### NPSC isolation, culture and treatment

NP tissues were collected from Sprague Dawley (SD) rat tail IVDs as previously reported [7]. The isolated NP tissues were digested with 0.2% collagenase type II (Sigma-Aldrich) in Dulbecco’s modified Eagle’s medium-low glucose (DMEM-LG) (HyClone) at 37 °C for 3 h. Then, the suspended cells were filtered through a 70-μm cell filter and centrifuged for 5 min at 1000 rpm/min. After removing the supernatant, the pellet was resuspended with DMEM-LG medium containing 10% foetal bovine serum (FBS) and 1% penicillin-streptomycin (HyClone). Subsequently, the isolated NP cells were cultured in a 25-cm^2^ cell-culture flask. After the first passage, the NPSCs were cultured in MethoCult H4230 methylcellulose medium (Stem Cell Technologies) as we have previously reported [14]. In brief, NP cells (total number of 10^3^ cells per dish) were cultured in 1 ml of MethoCult H4230 methylcellulose medium and seeded into 35-mm-diameter dishes for 2 weeks. Cell clusters (diameter greater than 50 μm) were isolated using a sterile Pasteur pipette and sub-cultured in a 25-cm^2^ cell-culture flask. All cell cultures were performed under a humidified atmosphere containing 5% CO_2_ at 37 °C. Passage 3 cells were used in this study.

Induction of NPSC quiescence by serum starvation: plate 2 × 10^5^ cells in a 25-cm^2^ cell-culture flask. NPSCs were expanded in growth medium (DMEM-LG containing 10%FBS) for 48 h. Proliferating cells (P) were sampled the day after seeding. After 48 h, wash the cells twice with 5 ml of PBS to completely remove the medium. And then add 5 ml of low-serum medium (DMEM-LG containing 0.1%FBS) to the plate. Finally, incubate the NPSCs in the incubator for 48 h or 3 weeks (depending on the experiments) and change medium (low serum) every 2 days.

Reactivation of quiescent NPSCs into a proliferating state: For restimulating serum-starved NPSCs, take a 25-cm^2^ cell-culture flask containing serum-starved cells and aspirate the low-serum medium. Then, add 5 ml growth medium (DMEM-LG containing 10%FBS) to the plate. Incubate the NPSCs in the incubator for 48 h. This process will re-stimulate serum-starved cells into a proliferating state. The reactivated NPSCs can be harvested by trypsinisation and re-plated on tissue culture plastic in growth medium for the following experiments such as colony-forming unit assay and trilineage differentiation assay.

Autophagy activation and inhibition: For autophagy activation in quiescent NPSCs, the proliferating cells were subjected to low-serum medium (DMEM-LG containing 0.1%FBS) containing rapamycin (1 μM, S1039, Selleck) for 48 h. For autophagy inhibition, the proliferating NPSCs were subjected to low-serum medium containing chloroquine (10 μM, S4157, Selleck) for 48 h.

### Identification of immunophenotypes

NPSCs were trypsinised, collected and washed twice with phosphate-buffered saline (PBS). Then, the NPSCs were stained with the following conjugated antibodies: CD29-PE (Biolegend), CD90-FITC (Biolegend), CD44-FITC (Biolegend), CD45-FITC (Santa Cruz) and CD34-FITC (Santa Cruz). An isotype control antibody (Biolegend) was used for each examined antibody. The final antibody concentration was 1 mg/100 mL. After incubating in the dark for 30 min at 37 °C, the cells were washed three times with PBS. Samples were subjected to flow cytometry (Beckman, USA), and the percentage of positive staining was calculated relative to the isotype control staining.

### Cell growth assay

The growth of NPSCs was assessed using a Cell Counting Kit-8 assay (CCK-8, Dojindo, Tokyo, Japan). Cells were plated in triplicate in 96-well tissue culture plates at a density of 1 × 10^4^ cells/cm^2^ in DMEM-LG medium containing 10% FBS. To assay cell proliferation at 1–7 days, the cells were washed twice with PBS and then incubated with 10 μl CCK-8 reagent for 2 h at 37 °C. The absorbance was measured at 450 nm using a microplate reader (Thermo Scientific, USA).

### Colony-forming unit (CFU) assay

To assess the self-renewal capacity of NPSCs, cells were trypsinised, and 1 × 10^2^ NPSCs per well were seeded in a 6-well cell culture cluster containing DMEM-LG medium. After 10 days of culture, the colonies were fixed with 4% paraformaldehyde, and then, Crystal Violet Staining Solution (Beyotime, Shanghai, China) was applied to stain the colonies. Colonies of more than 50 cells under the stereomicroscope were counted and analysed.

### Trilineage differentiation assay

We tested the multilineage differentiation of NPSCs by inducing the osteogenic, adipogenic and chondrogenic lineages in vitro. Briefly, the osteogenic differentiation of NPSCs was induced with osteogenic differentiation medium (Cyagen, Guangzhou, China) containing 10% FBS, 1% penicillin-streptomycin, 0.01% dexamethasone, 1% β-glycerophosphate and 0.2% ascorbate. After 3 weeks of induction, alizarin red staining (Cyagen) was used to identify mineral deposits. Cells were fixed with 4% formaldehyde at room temperature for 30 min and then stained with alizarin red solution (Cyagen) for 10 min at room temperature. Then, the cells were washed with PBS and microscopically observed. To quantify the stain, we used 2% cetylpyridinium chloride (Sigma-Aldrich) to elute the stain for 30 min and absorbance was measured at 540 nm.

Chondrogenic differentiation was induced by two micromass culture methods with different densities. The high-density method consisted of dropping 3 × 10^5^ cells in 15 μl into one well of a 24-well plate and allowing them to attach for 3 h in a 37 °C incubator. Then, the chondrogenic differentiation medium (Cyagen, Guangzhou, China) containing 0.01% dexamethasone, 0.3% ascorbate, 1% ITS + supplement, 0.1% sodium pyruvate, 0.1% proline and 1% TGF-β3 was added. After 3 weeks of induction, the micromass specimens were fixed with 4% formaldehyde and embedded in paraffin. Sections were cut at 4 μm and stained with alcian blue staining (Cyagen). The low-density method consisted of suspending NPSCs at 1 × 10^7^ cells per millilitre and spotting them as 15 μl drops into culture wells to induce chondrogenesis for 9 days. Subsequently, alcian blue staining was performed to assess the chondrogenic differentiation potential. Briefly, cells were rinsed with PBS, fixed with 4% paraformaldehyde for 15 min at room temperature, washed with PBS and then incubated in alcian blue solution for 2 h at room temperature. Then, the cultures were washed with PBS and observed with a microscope. To quantify the staining, we solubilised the stain in 6 M guanidine hydrochloride for 8 h at room temperature, and absorbance was measured at 600 nm.

Adipogenic differentiation was induced with adipogenic medium (Cyagen, Guangzhou, China), including medium A (10% FBS, 1% penicillin-streptomycin, 1% glutamine, 0.2% insulin, 0.1% IBMX, 0.1% rosiglitazone and 0.1% dexamethasone) and maintenance medium B (10% FBS, 1% penicillin-streptomycin, 1% glutamine and 0.2% insulin). After induction for 14 days, oil red O staining (Cyagen) was used for analysing adipogenesis. Cells were fixed with 4% formaldehyde solution for 30 min and then incubated in oil red O working solution for 30 min at room temperature. After washing 3 times with PBS, the cultures were observed with a microscope.

### Cell cycle analysis

NPSCs were trypsinised, collected and washed twice with PBS. Then, single cell resuspensions were fixed in pre-cooled 70% ethanol and stored at − 20 °C for later use. For cell cycle analysis, the fixed cells were centrifuged, washed twice with PBS containing 2% (*v*/*v*) FBS and resuspended in PBS. Subsequently, the resuspended cells were incubated in 500 μl staining solution containing Hoechst 33342 (for DNA content, 2 μg/mL) and Pyronin Y (for RNA content, 1 μg/mL) for 20 min at room temperature. Then, cells were placed on ice before flow cytometry analysis, and 10,000 events were collected per sample with FACS MoFlo (Beckman, USA).

### Immunofluorescent staining

Cells grown on coverslips were fixed in 4% paraformaldehyde for 15 min and permeabilised with 0.3% Triton X-100 for 15 min at room temperature. After 30 min of blocking with 3% BSA, the cells were incubated with a Ki67 antibody (Abcam) overnight at 4 °C and stained with secondary antibodies on a second day. Nuclei were counterstained with DAPI prior to imaging with a laser scanning confocal microscope (LSM, Carl Zeiss). The number of Ki67-positive cells was semiquantified by ImageJ.

### 5-Ethynyl-20-deoxyuridine (EdU) incorporation assay

Cell proliferation (DNA synthesis) was assessed by measuring EdU DNA incorporation using the Click-iT EdU Alexa Fluor 647 cell proliferation assay kit (RiboBio Co., Guangzhou, China). Briefly, cells were treated with EdU at 10 mg/ml for 48 h, washed in PBS/1% BSA and fixed with the Click-iT fixative. The cells were then permeabilized using a saponin-based permeabilisation reagent, treated with the Click-iT EdU reaction cocktail in the dark and washed with the saponin-based permeabilisation reagent. The number of EdU-positive cells was semiquantified by ImageJ.

### AD-mRFP-GFP-LC3 infection

First, NPSCs were plated and cultured in 15-mm glass bottom cell culture dishes. At a confluence of 30–50%, the cells were infected with AD-mRFP-GFP-LC3 (MOI 300, HB-AP07071804, Hanbio) according to the manufacturer’s instructions. Twenty-four hours after infection, the fresh complete medium was changed, and cells were viewed under a fluorescence microscope; > 95% of the cells was viable at that time. Then, the cells were administered different treatments. Images were captured with the aid of a laser scanning confocal microscope (LSM, Carl Zeiss). The green and red fluorescence (puncta) represented the phagophore and autolysosome, respectively, and the yellow fluorescence (puncta) represented the autophagosome. The number of red and yellow dots was determined by manually counting the fluorescent puncta in five high-power fields (× 60, LSM, Carl Zeiss).

### Western blotting

The protein extracts from NPSCs were harvested after lysing with a lysis buffer containing phosphatase and proteinase inhibitors (Beyotime, China). The total protein concentration was determined using the BCA protein assay reagent (Beyotime, China). The protein extracts were subjected to 10% or 12% SDS-PAGE. The protein was separated and then transferred to immunoblot PVD membranes (Millipore, Billerica, MA, USA). The membrane was blocked in 5% blotting-grade milk (Boster Biological Technology Co., Wuhan, China) in TBST for 1 h and then incubated with primary antibodies against P62 (Abcam), LC3 (CST), P27 (Proteintech) and GAPDH (Proteintech) in blocking solution overnight. After washing with TBST for 30 min, the membranes were incubated with the secondary antibody in blocking solution for 2 h at room temperature and then detected using a chemiluminescence assay (Millipore). Finally, the membranes were exposed to X-ray film to visualise the bands (Amersham Pharmacia Biotech). ImageJ was used to quantify the results.

### The siRNA preparation and transfection

To knockdown P27, a pool of siRNAs (RiboBio Co., Guangzhou, China) was tested for their capacity to silence P27. The target sequences were as follows. P27 siRNA-1: GGTCAATCATGAAGAACTA. P27 siRNA-2: GCAGATACGAGTGGCAGGA. The siRNA with the most potent silencing (P27 siRNA-1) was selected for the following experiments. Cells were re-plated and transfected with 200-μM siRNA at a confluence of 30–50%. Transfection was performed with the riboFECT™ CP reagent (RiboBio Co., Guangzhou, China) following the manufacturer’s instructions. After transfection for 24 h, the cells were returned to fresh growth media and administered different treatments. The effect of P27 silencing was detected by quantitative PCR and Western blotting analysis.

### Real-time quantitative PCR

The total RNA of cells was extracted using the TRIzol method. Total RNA (1 μg) was employed for reverse transcription using the Primescript RT reagent kit (Takara Bio, Shiga, Japan) according to the manufacturer’s protocols. Real-time quantitative PCR was performed on a ViiA™7 Real-time PCR system with SYBR® Premix Ex Taq™ II (Takara Bio, Shiga, Japan). GAPDH was used as an internal reference, and the level of the target gene was analysed using the 2^−∆∆Ct^ method, as described [15].

### Statistical analysis

The mean values obtained were compared by analysis of variance (ANOVA) with the SPSS13.0 software (SPSS). Data are presented as the mean ± standard deviation values. Student’s *t* test or one-way ANOVA was used for comparisons between groups; differences were considered statistically significant at *P* values < 0.05.

## Results

### Identification of rat NPSCs

**Fig. 1:**
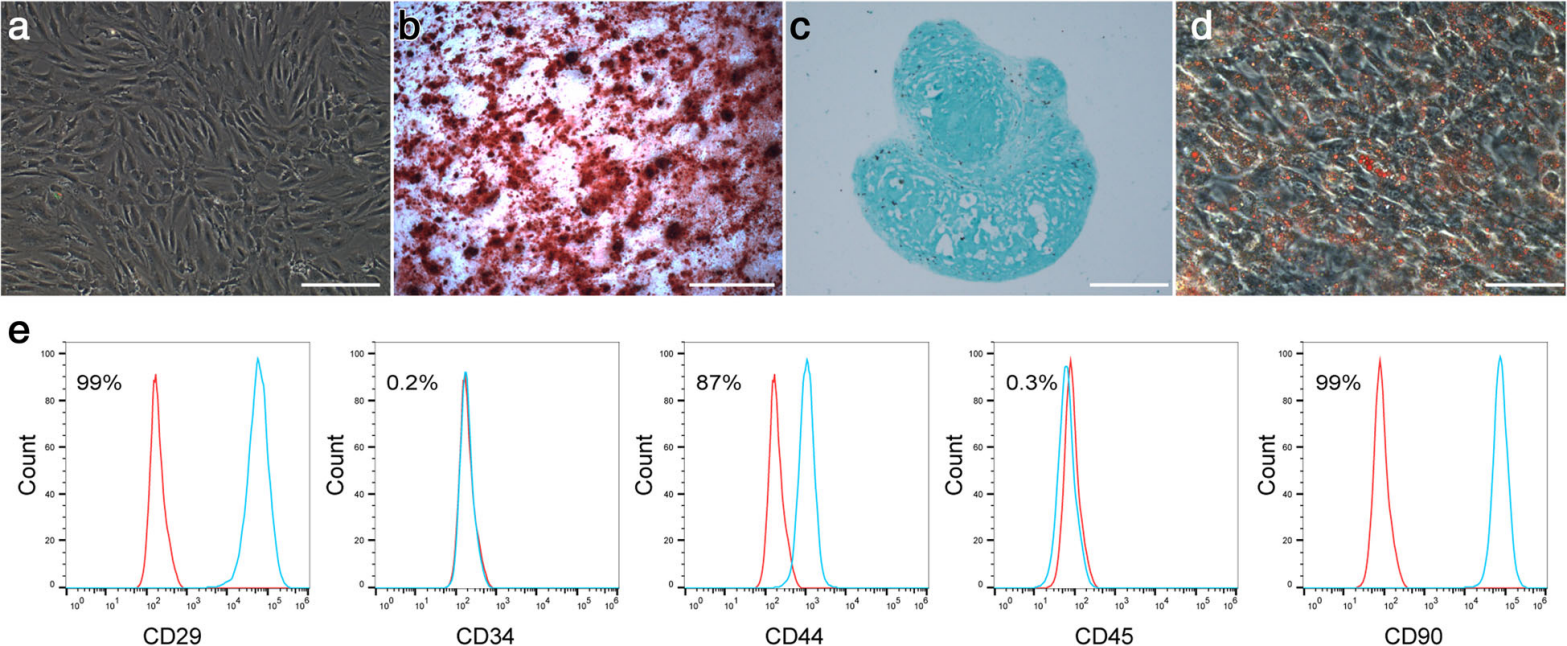
Isolation and identification of rat nucleus pulposus stem cells (NPSCs). **a** The purified NPSCs displayed a typical fibroblast-like morphology and a swirling-like pattern. **b** Alizarin red staining of NPSCs that underwent osteogenic induction for 3 weeks. **c** Histological section of chondrogenic microspheres formed by high-density micromass culture after 3 weeks stained with alcian blue. **d** Oil red O staining of NPSCs that underwent adipogenic induction for 3 weeks. **e** Identification of the immunophenotypic profile of stem cells by flow cytometric analysis. The green lines indicate the fluorescence intensity of cells stained with the corresponding antibodies, and the red lines represent the negative control cells. Scale bar = 200 μm

Cells from rat coccygeal IVD tissues exhibited a typical fibroblast-like morphology and a swirling-like pattern in monolayer culture, indicating the ability to adhere to plastic (Fig. 1a). The multilineage differentiation of NPSCs was evaluated by induction into the osteogenic (Fig. 1b), chondrogenic (Fig. 1c) and adipogenic (Fig. 1d) lineages in vitro. Based on the immunophenotype assays, the NPSCs were positive for stem cell markers, including CD29, CD44 and CD90 (Fig. 1e), but negative for CD34 and CD45 (Fig. 1e). In summary, the obtained cells exhibited features similar to those of multipotent mesenchymal stromal cells.

### An in vitro model for NPSC quiescence by serum starvation

**Fig. 2:**
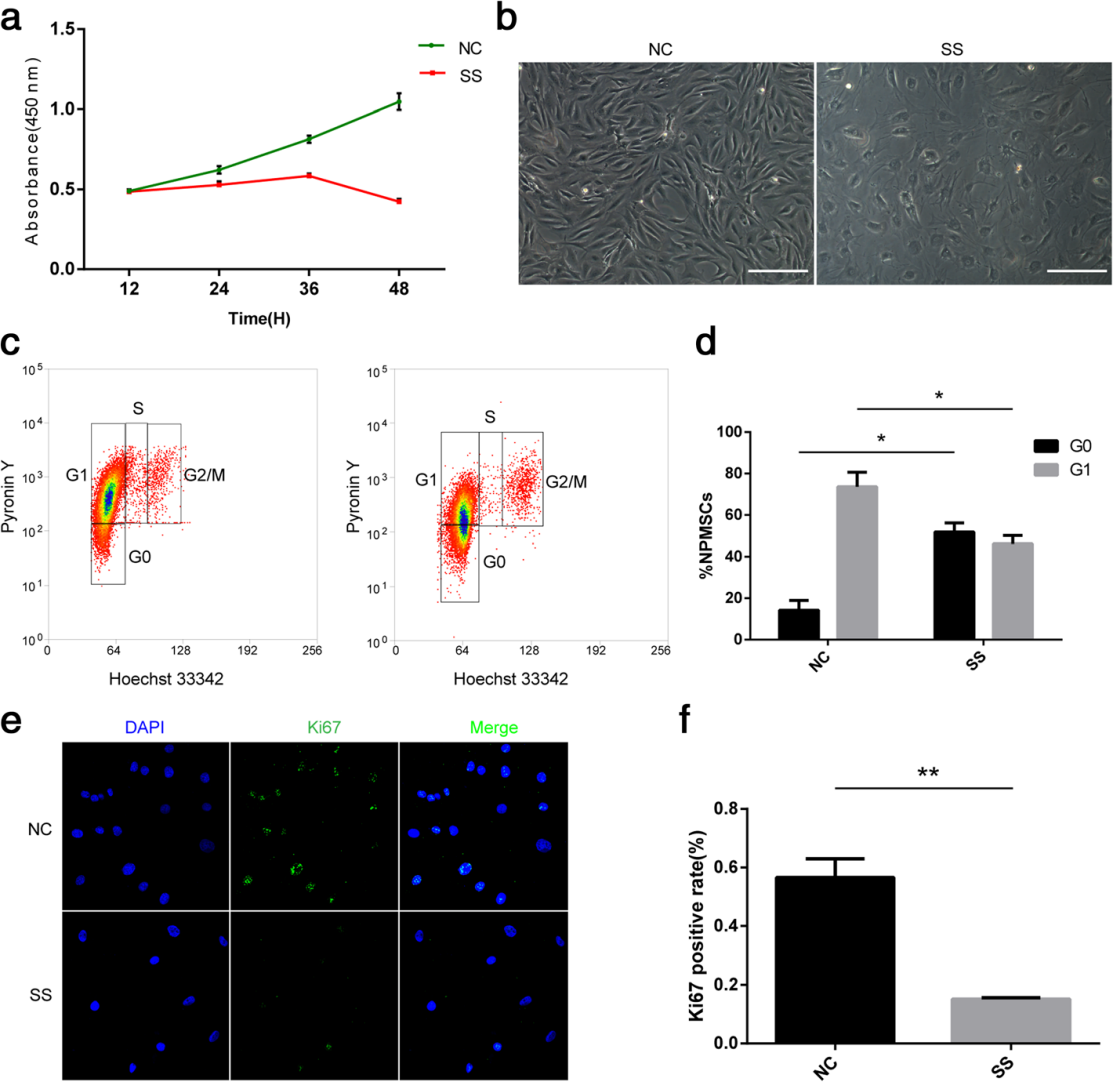
An in vitro model of cellular quiescence in NPSCs. **a** Cells were cultured under normal conditions (DMEM medium containing 10% serum) or serum starvation conditions (DMEM medium containing 0.1% serum), and growth was monitored at specified time intervals by CCK-8 assay. **b** Morphology of NPSCs cultured for 48 h under normal or serum starvation conditions. **c** Flow cytometry analysis of cells subjected to dual staining with Hoechst 33342 and Pyronin Y. The gated cells indicate cells in different phases of the cell cycle. **d** Under normal culture conditions, only approximately 14% of NPSCs were in the G0 phase with low RNA content as denoted by Pyronin Y staining, while serum starvation induced more than 51% of NPSCs to enter the G0 phase. The fraction of cells in the G1 phase decreased in the NPSCs induced into quiescence by serum starvation. Immunofluorescent staining of Ki67 (**e**) showed a significant decrease of 39% (from 54 to 15%) with serum starvation (**f**). Scale bar = 200 μm. The results are presented as the mean ± standard deviation, *n* = 3. *p < 0.05, **p < 0.01. NC normal conditions (DMEM medium containing 10% serum), SS serum starvation conditions (DMEM medium containing 0.1% serum), H hours, DAPI 4′,6-diamidino-2-phenylindole

A variety of in vitro models of quiescence in different cell types have been established under well-controlled experimental conditions (e.g., mitogen removal). To generate quiescent NPSCs, the cells were cultured in a medium containing 0.1% FBS that has previously been described to induce quiescence in primary fibroblasts [4]. Growth kinetics were evaluated by a CCK-8 assay, which showed a maximal growth inhibition at 48 h for cells grown in the low-serum condition (Fig. 2a). After 48 h of culture, the NPSCs treated with serum starvation (0.1% FBS) became shrunken and round in morphology (Fig. 2b). We next determined the cell cycle states by flow cytometry analyses of dual staining with Hoechst 33342 and Pyronin Y dye, a standard cell cycle analysis method capable of distinguishing G1 and G0 states (Fig. 2c). After 48 h of treatment, while under normal growth conditions (10% FBS), only approximately 14% of NPSCs were in the G0 state; however, serum starvation induced more than 51% of NPSCs to enter the G0 state (Fig. 2c, d). In addition, we investigated the expression rate of the proliferation marker Ki67, which is present during all active phases of the cell cycle except G0. Consistent with the cell cycle analysis, Ki67 staining showed a significant decrease with serum starvation (Fig. 2e, f).

### Induction of quiescence protects NPSC function

**Fig. 3:**
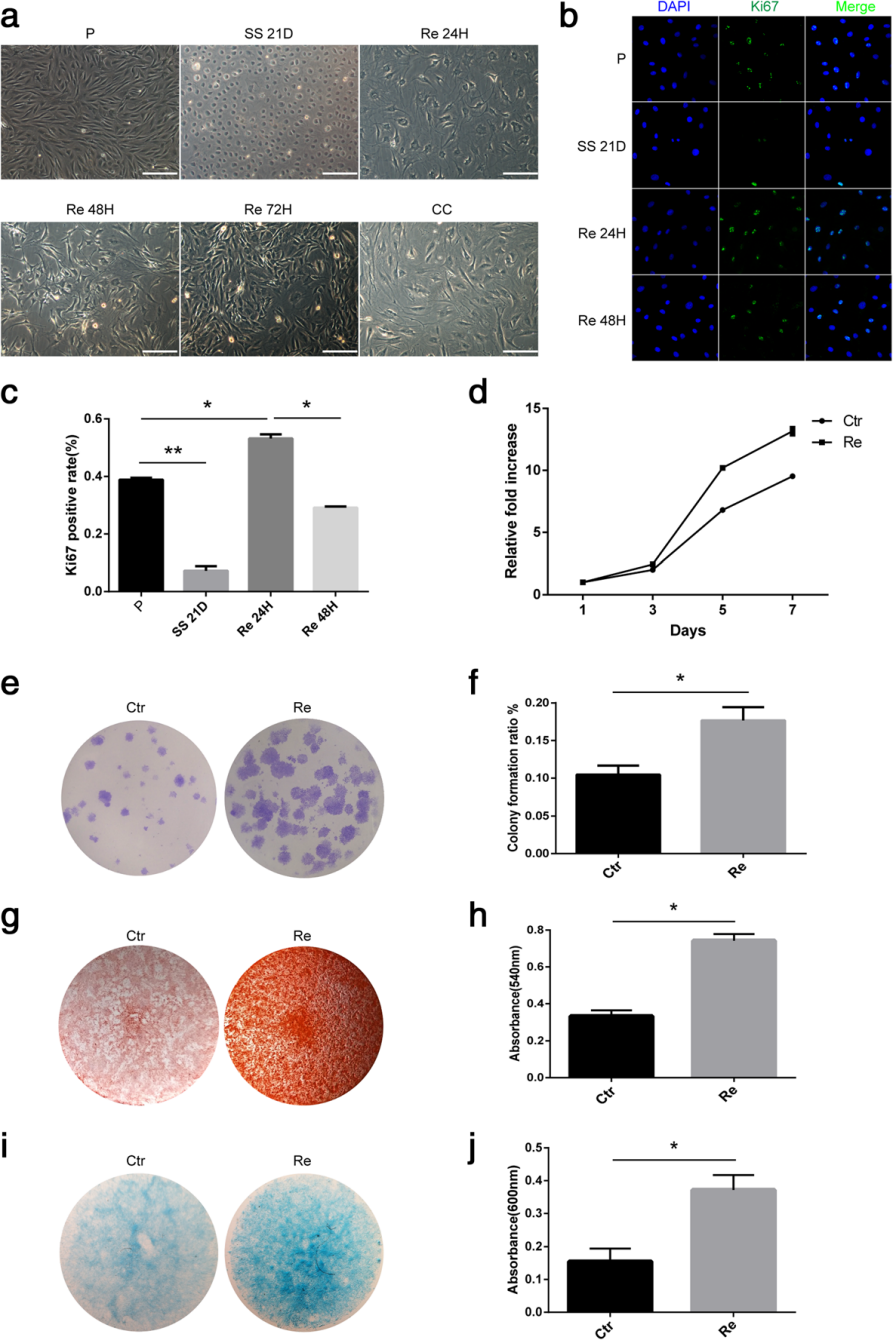
Assessment of function of reactivated NPSCs after serum starvation for 3 weeks. **a** Morphology of NPSCs during proliferation (P), serum starvation for 21 days (SS 21D), reactivation for 24 h, 48 h and 72 h after serum starvation (Re 24H, Re 48H and Re 72H) and control cultures (CC). **b**, **c** Ki67 staining and detection of the percent of NPSCs positive for Ki67 during proliferation (P), serum starvation for 21 days (SS 21D), reactivation for 24 h, 48 h and 72 h after serum starvation (Re 24H, Re 48H and Re 72H) and control cultures (CC). **d** NPSCs from reactivation and control groups were seeded at 1 × 10^4^ cells/cm^2^ and cultured for 7 days. CCK-8 assay was performed during the indicated period, and the data are shown as the relative fold increase. **e**, **f** CFU staining and quantitative analysis of the NPSCs from control and reactivation groups. **g**, **h** Alizarin red staining and quantitative analysis of the NPSCs from the control group and reactivation group. **i**, **j** Alcian blue staining and quantitative analysis of the NPSCs from both groups. Scale bar = 200 μm. All data are expressed as the mean ± standard deviation. *n* = 3. *p < 0.05, **p < 0.01. Ctr control group, Re reactivation group, DAPI 4′,6-diamidino-2-phenylindole

To further understand the biology of quiescent NPSCs, incubation with serum starvation continued for 3 weeks. To determine whether cell cycle arrest is reversible, the cells of reactivation group (Re) were re-stimulated with 10% serum after serum starvation for 3 weeks (see the “Methods” section for details). Cells from the control group (Ctr) were incubated in medium containing 10% FBS, and the adherent cells were sub-cultured when they reached 80–90% confluence. Additionally, the cells were stained with the proliferation marker Ki67 (Fig. 3b). The morphology of the serum-stimulated cells changed from small, round cells to the spindle-shaped cell characteristic of early-passage NPSCs (Fig. 3a). However, the NPSCs cultured with the normal 10% serum became large, apparently senescent cells (Fig. 3a). Moreover, the re-stimulated NPSCs re-entered the cell cycle as evidenced by increased cells stained for Ki67, with the number of labelled cells peaking at 24 h (Fig. 3c). In addition, we found that the reactivated cells had a significantly increased growth rate compared with normally cultured cells (Fig. 3d).

To examine whether the induction of G0 in NPSCs affects their self-renewal, we determined the colony-forming capacity after the induction of G0 with the CFU assay (Fig. 3e). The reactivated G0 NPSCs exhibited a significantly increased number of CFU compared to the control cultures (Fig. 3f).

To address whether the induction of G0 affects the differentiation capacity of NPSCs, we assessed the osteogenic and chondrogenic differentiation potential of the post-G0 reactivated NPSCs. The osteogenic differentiation capacity of the reactivated NPSCs was significantly increased compared with that of the normal cultures; this was evident from alizarin red staining (Fig. 3g, h). Alcian blue staining showed that the reactivated NPSCs also maintained chondrogenic differentiation capacity compared with control cultures (Fig. 3i, j).

### Autophagy mediates the quiescence of NPSCs and regulates the expression of P27

**Fig. 4:**
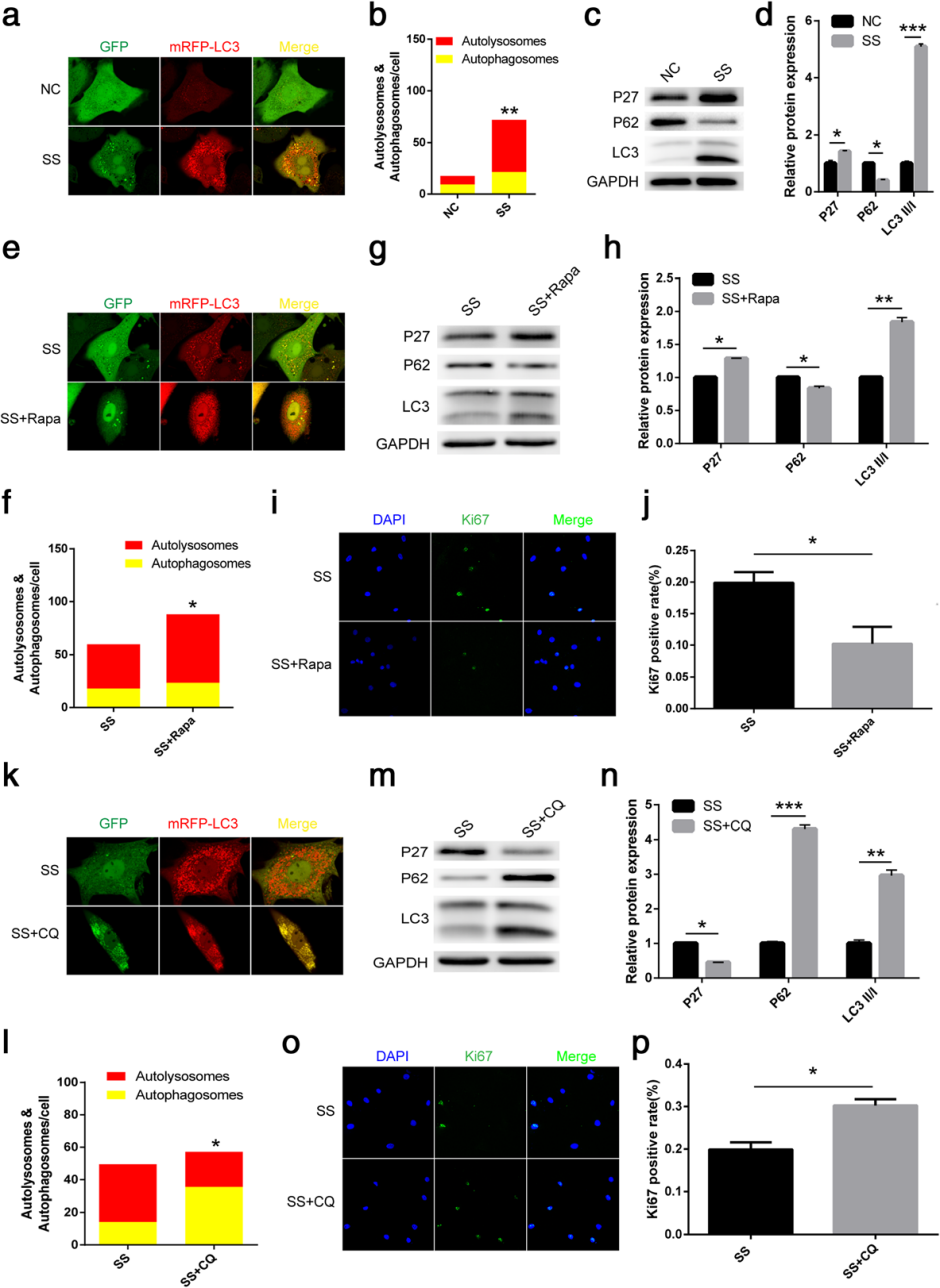
Autophagy mediates the quiescence of NPSCs and regulates the expression of P27. (1) Quiescent NPSCs induced by serum starvation upregulate autophagy and the expression of P27. Representative images of GFP and mRFP puncta under normal and serum starvation conditions are shown (**a**), together with the quantification of autophagosomes and autolysosomes (**b**). *n* = 5. **c**, **d** Western blot analysis was performed to examine the expression of P27, LC3 and P62 at the protein level. GAPDH served as an internal control. Data are expressed as the mean ± standard deviation, *n* = 3. *p < 0.05, **p < 0.01, ***p < 0.001. NC normal culture condition, SS serum starvation condition. (2) Autophagy promotes NPSC quiescence. Representative images of GFP and mRFP puncta under serum starvation conditions with or without rapamycin are shown (**e**), together with the quantification of autophagosomes and autolysosomes (**f**). *n* = 5. **g**, **h** Western blot analysis was performed to examine the expression of P27, LC3 and P62 under serum starvation conditions with or without rapamycin. **i**, **j** Ki67 staining showed that NPSC proliferation was further inhibited after 48 h of rapamycin treatment under serum starvation conditions. Data are expressed as the mean ± standard deviation, *n* = 3. *p < 0.05, **p < 0.01. SS serum starvation condition, Rapa rapamycin. (3) autophagy inhibition disturbs the quiescence of NPSCs. Representative images of GFP and mRFP puncta under serum starvation conditions with or without chloroquine are shown (**k**), together with the quantification of autophagosomes and autolysosomes (**l**). *n* = 5. **m**, **n** Western blot analysis was performed to examine the expression of P27, LC3 and P62 under serum starvation conditions with or without chloroquine. **o**, **p** Ki67 staining showed that NPSC proliferation increased after 48 h of chloroquine treatment under serum starvation conditions. Data are expressed as the mean ± standard deviation, *n* = 3. *p < 0.05, **p < 0.01, ***p < 0.001. SS serum starvation condition, CQ chloroquine, DAPI 4′,6-diamidino-2-phenylindole

Basal autophagy is essential to maintain a quiescent state in many other cells, such as satellite cells and glioblastoma cells. We wondered whether and how autophagy was involved in the regulation of quiescence in NPSCs. We employed the AD-mRFP-GFP-LC3 reporter and analysed autophagy under normal and serum starvation conditions. Twenty-four hours after infection, cells were switched to either normal or serum starvation conditions. The formation of autophagosomes and autolysosomes, as indicated by the yellow (emission of both green and red fluorescence) and red puncta, respectively, was visibly enhanced with serum starvation for 48 h (Fig. 4a, b). We analysed the protein expression of the key autophagy-associated proteins P62 and LC3II/I in two culture conditions with a Western blot. Consistent with the reporter analyses, serum starvation significantly induced the expression of the LC3II/I protein and reduced the expression of the P62 protein compared with the levels of these proteins in normal cells (Fig. 4c, d). Previously, Lemons et al. [4] showed the induction of P27 as cells entered the state of quiescence. Cells under serum starvation for 48 h showed a significantly increased level of p27 as evidenced by Western blot analysis, indicative of quiescence-like growth arrest (Fig. 4c, d).

To investigate whether autophagy underlies serum starvation-induced NPSC quiescence, we subjected the cells to serum starvation with or without rapamycin (1 μM), an autophagy agonist for 48 h. As expected, serum starvation with rapamycin treatment induced more red puncta (autolysosomes) than no rapamycin treatment, indicating that autophagy was enhanced (Fig. 4e, f). In addition, a significant reduction in P62 protein expression and an upregulation of the proteins LC3II/I and P27 were also observed in cells under serum starvation with rapamycin treatment compared with those under serum starvation without rapamycin as determined by Western blot analysis (Fig. 4g, h). These results illustrated that rapamycin further enhanced autophagic activity in serum-starved NPSCs, and rapamycin also enhanced the entrance of NPSCs into quiescence by increasing the level of the protein P27 (Fig. 4g, h). In addition, we also determined that enhanced autophagy drives cells into quiescence with Ki67 staining. The rapamycin-treated group showed a significantly lower proportion of cells positive for Ki67 than the control group, indicating the inhibition of proliferation (Fig. 4i, j).

To further confirm the effect of autophagy on NPSC quiescence, we inhibited autophagy activity when the induced cells entered G0 by the serum starvation treatment. Therefore, we treated NPSCs with serum starvation conditions with or without chloroquine (10 μM), an autophagy inhibitor (lysosomal acidification inhibitor), for 48 h. As expected, chloroquine treatment induced more yellow autophagosome puncta and less red autolysosome puncta, indicating reduced autophagy (Fig. 4k, l). In addition, Western blot analysis showed that the addition of chloroquine significantly induced both the expression of the LC3II/I and P62 proteins but reduced the expression of the P27 protein (Fig. 4m, n). As such, these results illustrated that chloroquine inhibited autophagic activity in serum-starved NPSCs; importantly, chloroquine also prevented NPSCs from entering quiescence by decreasing the level of the protein P27 (Fig. 4m, n). Consistent with the Western blot analyses, the addition of chloroquine increased cell proliferation by 1.5 times under serum starvation conditions as evidenced by Ki67 staining (Fig. 4o, p).

### P27 siRNAs prevent the entrance of NPSCs into quiescence

**Fig. 5:**
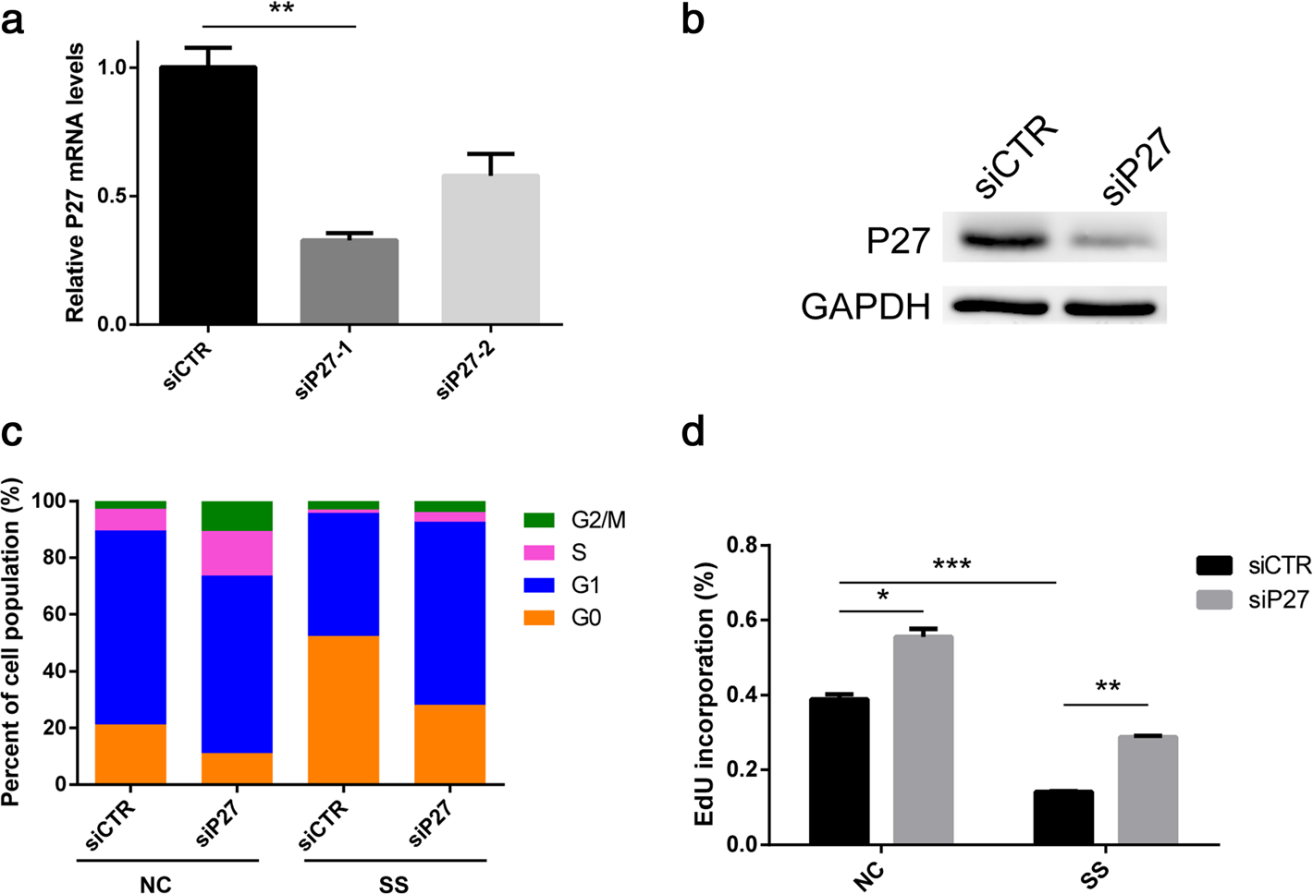
P27 siRNAs prevent the entrance of NPSCs into quiescence. **a** The quantitative PCR analysis of P27 mRNA expression was performed using NPSCs transfected with P27 siRNA (siP27) and a scrambled negative control siRNA (siCTR). **b** Representative Western blot analysis showed a reduction of P27 following the transfection of NPSCs with p27 siRNA. **c** The transfected NPSCs were stained with Pyronin Y and Hoechst 33342 and analysed by flow cytometry under normal and serum-starved conditions. **d** Detection of EdU incorporation in transfected NPSCs under both conditions. Data are expressed as the mean ± standard deviation, *n* = 3. *p < 0.05, **p < 0.01, ***p < 0.001. NC normal culture condition, SS serum starvation condition

According to the results described above, the protein expression of P27 not only increased in quiescent NPSCs but could also be regulated by autophagy. We wondered whether p27 is required for NPSC quiescence as in other cell types. Therefore, P27 siRNAs were constructed and transfected into NPSCs. The endogenous P27 mRNA levels in NPSCs significantly decreased after transfection with P27 siRNAs. Sequence-1 P27 siRNA (P27 siRNA-1) was selected for the following experiment (Fig. 5a). Western blot analysis also demonstrated that P27 expression was decreased in P27 siRNA transfected NPSCs (Fig. 5b). Next, we assessed the role of P27 siRNA in NPSC quiescence by flow cytometry analyses and an EdU incorporation assay under normal and serum starvation conditions. Both the cell cycle and EdU incorporation analyses showed that proliferation was inhibited under serum-starvation; however, P27 siRNA reduced the percentage of the population in the G0 state and increased EdU incorporation in both the normal and serum-starved conditions (Fig. 5c, d). These experiments demonstrate that P27 siRNA enhances proliferation and prevents NPSCs from entering quiescence.

## Discussion

Cellular quiescence, a reversible mode of exiting the cell cycle, is a novel area of research within the field of stem cell biology. As a critical characteristic of adult stem cells in vivo, quiescence maintains the capacity for cell cycle re-entry and subsequent differentiation in response to environmental stimuli [5, 9, 16]. Several studies have proposed the existence of stem cells in human, mouse, rat, rabbit, porcine, canine, bovine and rhesus monkey nucleus pulposus tissues [7, 17–21]. We also successfully isolated and identified NPSCs in rat nucleus pulposus tissue (Fig. 1). However, recent studies demonstrated that the number and functions of NPSCs declined with age due to cellular senescence [7, 8]. Given that previous studies suggested cellular quiescence maintains stemness and prevents premature exhaustion and senescence of stem cells, we hypothesise that quiescence may play a role in age-related changes in NPSCs. However, an explicit analysis of G0 NPSCs has not been reported. Here, as a first step in understanding the biology of quiescent NPSCs, we established a model for NPSC quiescence by serum starvation in vitro.

Mammalian cells can be induced to enter a quiescent state by a variety of in vitro models, including anchorage deprivation, serum starvation, contact inhibition, suspension culture and culture on soft polyacrylamide substrate. However, as noted by Rumman [16], a key consideration concerns the type of growth arrest attained by the abrogation of mitogenic signalling in different cell types. For example, contact inhibition is a well-established model for cellular quiescence in primary fibroblasts retinal pigment epithelium cells [4, 22]. However, this model may not be suitable for human bone marrow MSCs (hBMSCs) because contact inhibition is inefficient in suppressing hBMSC proliferation [23]. Considering the avascular structure of nucleus pulposus tissue, we chose serum starvation to induce NPSC quiescence in vitro. We are pleased that a protocol of serum starvation for 48 h proved effective in inducing NPSC quiescence. To further confirm the reliability of this model, we kept NPSCs under the serum-starved condition for 21 days. Next, we observed the properties of the NPSC progenitor cells after re-stimulation with 10% serum.

Reversibility is a defining characteristic of cellular quiescence: in contrast to other non-dividing cells, such as terminally differentiated cells and senescent cells, only quiescent cells retain the ability to re-enter the cell cycle in response to normal physiological stimuli [9]. Our experiments also confirmed this finding: after serum starvation for 21 days, NPSCs stimulated with serum resumed their proliferation. Coller [24] proposed a new description of cellular quiescence that one mechanism in which the reversibility of quiescence is insured by the suppression of terminal differentiation. A recent report also suggested that the preservation of quiescence protects muscle stem cell function [3]. Therefore, we further examined the self-renewal abilities and differentiation potential of reactivated NPSCs. All results support that the induction of quiescence maintains the properties of NPSCs. It is not difficult to understand why previous reports have associated the loss of quiescence with accelerated ageing and pathological cellular changes. Therefore, we have a reason to infer the role of quiescence in NPSC senescence. It is especially important to explore the mechanisms that maintain NPSC quiescence.

Autophagy, a homeostatic ‘clean up’ process, is particularly critical in non-dividing stem cells, in which the mitotic dilution of intracellular toxic debris does not take place. In the absence of autophagy, long-lived, non-dividing quiescent stem cells may accumulate damage from environmental stress [9]. In the skeletal muscle, autophagy is a key factor in maintaining the regenerative capacity of muscle stem cells by promoting quiescence and preventing senescence [13]. Human clinical trials and animal model studies have also documented that autophagy significantly contributes to IVD degeneration [25–27]. Similarly, we also detected the activation of autophagy in NPSCs induced into quiescence by serum starvation. Under environmental stresses, the induction of autophagy seems to be important in the regulation of stem cell activation [9]. For example, autophagy regulates the entry of glioblastoma cells and ovarian cancer spheroid cells into the quiescent state [10, 28]. However, the detailed mechanism of autophagy in the regulation of quiescence remains to be fully elucidated. In our experiments, we also examined the entry into the quiescent state by activating and inhibiting autophagy. Intriguingly, we observed that the expression of the cyclin-dependent kinase inhibitor P27 increased in quiescent NPSCs. During the process of inducing quiescence, rapamycin treatment significantly increased the level of P27, while chloroquine treatment decreased the level of P27. Thus, we speculate that autophagy regulates NPSC quiescence by regulating the expression of P27.

The molecular mechanisms of adult stem cell quiescence involve both cell-intrinsic factors, such as the P53 and RB proteins and CDK inhibitors (P21, P27 and P57), and cell-extrinsic factors, such as Notch signalling and Wnt signalling [16]. Cellular quiescence markers differ in different cell types. P27 has been proven to maintain quiescence in some cell types, such as fibroblasts, radial stem cells and haematopoietic stem cells [29–31]. Whether P27 maintains the quiescence of NPSCs is largely unknown. To investigate the specific contribution of p27 in the control of serum starvation-induced G0 arrest, we specifically reduced its synthesis by the transfection of short interfering RNAs. The knockdown of p27 reduced the percentage of G0 cells and enhanced the proliferation of NPSCs. These results suggested that p27 is required for NPSCs to enter a quiescent state. Therefore, we confirm the above inference that autophagy regulates NPSC quiescence by regulating the expression of P27.

This study also has some limitations. We confirmed the existence of quiescence in NPSCs by serum starvation in vitro. Furthermore, the conclusion was also based on in vitro evidence. There is no precise animal model to examine quiescence and its regulatory mechanism in NPSCs in vivo. Although in vitro models provide a simple, readily scalable and reproducible system for the analysis of quiescence, they do lack the complexity of the in vivo environment. Further studies are required to assess how closely the molecular phenotype can be extrapolated to NPSCs in vivo. In addition, although the positive effect of autophagy on quiescence was shown, the exact mechanism is still unclear and requires further study.

## Conclusions

In conclusion, serum starvation efficiently induces quiescence in NPSCs. The induction of quiescence in NPSCs maintains stem cell properties, including clonogenic self-renewal, osteogenic differentiation and chondrogenic differentiation. Our study reveals that autophagy plays a role in maintaining NPSC quiescence and demonstrates that autophagy mediates the quiescence of NPSCs by regulating P27. These findings are relevant for future studies addressing aspects of the in vivo biology of NPSCs and provide novel insights into the decline of function in endogenous NPSCs during ageing and degeneration of IVD.

## Abbreviations

ANOVA: Analysis of variance
CCK-8: Cell Counting Kit-8
CFU: Colony-forming unit
CQ: Chloroquine
Ctr: Control group
DAPI: 4′,6-Diamidino-2-phenylindole
DMEM-LG: Dulbecco’s modified Eagle’s medium-low glucose
EdU: 5-Ethynyl-20-deoxyuridine
FBS: Foetal bovine serum
hBMSCs: Human bone marrow mesenchymal stem cells
IDD: Intervertebral disc degeneration
IVD: Intervertebral disc
MSC: Mesenchymal stem cell
NC: Normal culture condition
NPSC: Nucleus pulposus stem cell
PBS: Phosphate-buffered saline
Rapa: Rapamycin
Re: Reactivation group
SS: Serum starvation conditions
